# Splenic Rupture: A Delayed and Rare Complication of Colonoscopy

**DOI:** 10.7759/cureus.112076

**Published:** 2026-07-05

**Authors:** Samrudh Shaju

**Affiliations:** 1 Emergency Medicine, Caboolture Hospital, Brisbane, AUS

**Keywords:** colonoscopy complications, post-procedure complication, spleen hemorrhage, spleen rupture, splenic trauma

## Abstract

Splenic rupture is an extremely rare but significant complication of colonoscopy with a high mortality rate. Most patients present within 24 to 48 hours post-procedure with abdominal pain, hypotension, and/or syncope. This case report highlights the rare case of a patient presenting nine days post-procedure with splenic rupture. This case highlights the importance of having a high index of suspicion for splenic rupture for up to two weeks after colonoscopy, as a delay in diagnosis can cause rapid clinical deterioration and death. Early diagnosis of splenic rupture can be achieved with CT of the abdomen, which remains the gold standard. Patients should also be made aware of this potentially delayed complication and be advised to monitor for significant abdominal pain for up to two weeks after colonoscopy to ensure prompt management.

## Introduction

Colonoscopy is a diagnostic and therapeutic procedure that is very well tolerated in the general population. Complications are rare and include bowel perforation (0.34% to 2.14%) and gastrointestinal haemorrhage (1.8% to 2.5%) [1]. Splenic rupture is an extremely rare complication with an incidence of 0.00005% to 0.017% and a mortality rate of 5% [2]. It is presumed to be caused by significant splenocolic ligament traction and/or direct trauma to the spleen during the procedure due to difficult navigation [3]. Intraperitoneal adhesions or any previous underlying splenic pathology (e.g. lesions, congenital anomalies) may increase the incidence and risk of splenic rupture as a complication of colonoscopy [4]. Patients most commonly present with nonspecific abdominal pain, hypotension, and/or syncope within 24-48 hours post-procedure [5]. A delayed presentation beyond this window is quite rare, but case reports have described patients presenting up to two weeks post-procedure with a splenic rupture [6][7]. It is hypothesised that delayed presentations are due to further straining of the perisplenic ligament post-procedure through physical activity, ultimately resulting in ligament or capsular injury [8]. However, the exact mechanism remains unclear. This case report highlights another unusual case of delayed presentation of splenic rupture in a 46-year-old female nine days after colonoscopy.

## Case presentation

A 46-year-old female presented to the emergency department with sudden-onset left upper quadrant (LUQ) abdominal pain at 1300 hours while doing administrative work at a computer. The pain was described as sharp, colicky, and rated as 6/10. The pain was initially localised to the abdomen but worsened to 8/10 and began radiating to the left shoulder and scapula at 1700 hours. She reported a syncopal episode in the shower due to the pain, prompting her visit to the emergency department. She had undergone a colonoscopy nine days earlier for investigation of a positive faecal occult blood test. She reported no interventions during the procedure or complications post-procedure. The colonoscopy report showed no complications or difficulties with navigation during the procedure. She denied any bowel or urinary incontinence. She also denied any nausea, vomiting, subjective fevers, or coryzal symptoms. She had a past medical history of anxiety (managed with duloxetine) and gastro-oesophageal reflux disease (managed with esomeprazole). She had also commenced a GLP-1 agonist for weight loss five months earlier but reported no nausea, vomiting, or any other gastrointestinal complications. The patient had a surgical history of stomach intestinal pylorus-preserving surgery (SIPS) in 2019 and laparoscopic appendicectomy in 2006, suggesting an increased risk of intra-abdominal adhesions and, thus, an increased risk of splenocolic ligament traction during colonoscopy.

Clinical examination revealed a tympanic temperature of 36.6°C and a blood pressure of 89/60 mmHg with a heart rate of 90 beats/minute. Fluid resuscitation was commenced immediately, and the blood pressure responded to fluids, improving to 129/70 mmHg after a 1 L bolus of sodium chloride. The abdomen was soft, but the patient had significant tenderness in the LUQ with voluntary guarding. The patient also reported radiating pain to the left scapula when lying flat, which was consistent with Kehr's sign. A full blood count, serum chemistry, and group and save were collected, which showed a reduced haemoglobin level of 112 g/L and mild leucocytosis of 16.5 × 10⁹/L (Table 1). Given concerns of an acute abdomen following a recent procedure, a contrast-enhanced CT of the abdomen and pelvis was performed within an hour of presentation, which showed a splenic haematoma with features of ongoing active haemorrhage and extracapsular extension (Figure 1). The patient was urgently transferred to a tertiary hospital with interventional radiology capabilities. A CT angiogram of the abdomen was performed at the receiving hospital (four hours after presentation), which showed progression of the splenic haemorrhage and an arterial blush at the inferior edge of the spleen (Figure 2). Interventional radiology performed distal embolisation overnight, but the patient subsequently developed haemodynamic instability on the ward with a haemoglobin drop to 79 g/L, requiring laparotomy, splenectomy, and transfer to the ICU post-procedure. The patient was commenced on amoxicillin for antibiotic prophylaxis and received pneumococcal conjugate, meningococcal conjugate, meningococcal B recombinant, and Haemophilus influenzae type b conjugate vaccines. She also required two units of packed red cells postoperatively. After two days in the ICU, she was stepped down to the surgical ward for 24 hours of observation and then discharged with a post-splenectomy information sheet and planned GP follow-up for antibiotic prophylaxis for three years and ongoing regular vaccinations according to a post-splenectomy vaccination schedule. She was also scheduled for follow-up in the general surgical clinic to review clinical symptoms four weeks post-procedure.

**Table 1:** Key full blood examination parameters for the patient

Parameters (units)	Patient Values	Reference
Haemoglobin (g/L)	112	115-165
White cell count (×10^9^)	16.5	3.5-11.0
Neutrophils (×10^9^)	14.80	1.7-7.0

**Figure 1:**
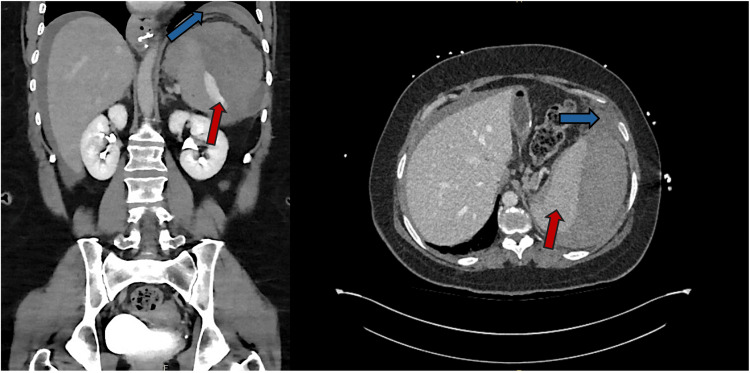
Contrast-enhanced CT of the abdomen showing coronal (left) and axial (right) views of a splenic haemorrhage (red arrows) and a perisplenic haematoma (blue arrows).

**Figure 2:**
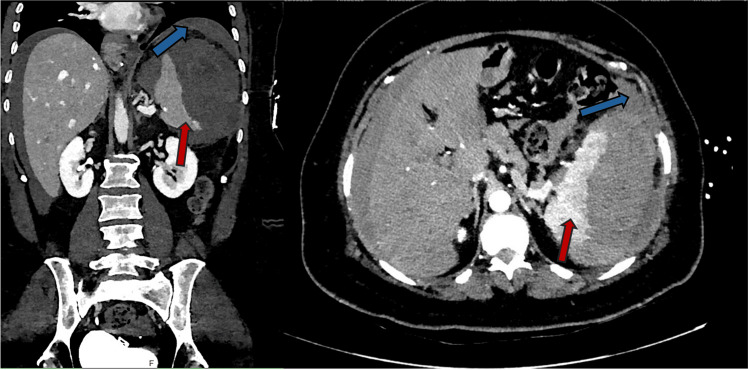
CT angiogram of the abdomen showing coronal (left) and axial (right) views demonstrating progression of the splenic haemorrhage (red arrows) and perisplenic haematoma (blue arrows).

## Discussion

Splenic rupture is an extremely rare complication of colonoscopy with a high mortality rate that normally presents within 24-48 hours post-procedure. This case report highlights an unusually delayed presentation nine days post-procedure in a 46-year-old female. The patient's clinical presentation of abdominal pain, hypotension, and syncope is consistent with previous cases of splenic rupture [5]. She also had an increased risk of splenocolic traction from intra-abdominal adhesions given her previous history of abdominal surgery. However, her colonoscopy report showed no difficulties with navigation or any other complications. An additional case has been reported in the literature in which the patient presented 13 days post-procedure [1]. This suggests that significant abdominal pain with or without associated hypotension and/or syncope, even two weeks after colonoscopy, should be met with a high index of suspicion for splenic rupture given the high risk of mortality. Additionally, risk factors such as mechanical difficulties during the procedure or previous intra-abdominal surgery should prompt the clinician to consider splenic rupture as the primary diagnosis. CT of the abdomen is the gold standard for diagnosing splenic rupture or haemorrhage promptly, providing detailed imaging of the splenic parenchyma, vascular structures, and any associated abdominal injury [8]. This study acknowledges the inherent limitations of a case report and its inability to support broad diagnostic recommendations. Therefore, the above suggestions should be considered only after a thorough clinical history, examination, investigations, and application of sound clinical judgement by the clinician.

## Conclusions

In conclusion, it is suggested that significant abdominal pain with haemodynamic instability and/or syncope within two weeks of colonoscopy should prompt the clinician to strongly consider splenic rupture in the differential diagnosis and, thus, obtain a prompt CT of the abdomen. This is especially true if the patient has risk factors for splenic rupture (i.e., previous abdominal surgery or splenic pathology) or if the colonoscopy procedure note reported prolonged or difficult navigation. Patients should also be made aware of this rare potential complication given its high mortality risk. They should be advised to present promptly to the emergency department if experiencing significant abdominal pain with or without syncope for up to two weeks post-procedure, given multiple reported cases of delayed presentation of splenic rupture in the current literature. Given the limitations of individual case reports, the above recommendations should be considered in conjunction with thorough history taking, clinical examination, and sound clinical judgement.
